# Near complete response after single dose of nivolumab in patient with advanced heavily pre-treated *KRAS* mutant pulmonary adenocarcinoma

**DOI:** 10.1186/s40164-015-0029-7

**Published:** 2015-12-14

**Authors:** Diwakar Davar, Mark A. Socinski, Sanja Dacic, Timothy F. Burns

**Affiliations:** Division of Hematology-Oncology, Department of Medicine, University of Pittsburgh Medical Center, Pittsburgh, PA USA; Division of Anatomic Pathology, University of Pittsburgh Medical Center, 200 Lothrop Street, Office: PUH C608, Pittsburgh, PA 15213 USA; Division of Hematology-Oncology, University of Pittsburgh Cancer Institute, 5150 Centre Avenue, Office: Room 556, 5th floor, Pittsburgh, PA 15232 USA; Division of Hematology-Oncology, University of Pittsburgh Cancer Institute, 5117 Centre Avenue, Office: Suite 2.18e, Pittsburgh, PA 15232 USA

**Keywords:** Lung cancer, Adenocarcinoma, *KRAS* mutant, Nivolumab, Programmed death receptor-1 (PD-1)

## Abstract

The programmed death 1 (PD-1) receptor is expressed by activated T-cells and engaged by ligands PD-L1 and PD-L2 normally expressed by infiltrating immune cells in response to viral infection. The PD-1/PD-L1 axis is a negative inhibitory pathway that down-regulates T-cells but is also used by tumors to evade anti-tumor immunity. Antibodies targeting PD-1/PD-L1 axis are capable of restoring functional anti-tumor immunity and have demonstrated efficacy in a broad range of tumor types including non-small cell lung cancer in both squamous and adenocarcinoma histologies. Ongoing issues affecting clinical development of these agents include assessment of response, optimal duration of therapy in excellent responders, predictive biomarkers and mechanisms of resistance. In this report, we describe a patient with advanced KRAS mutant heavily pretreated pulmonary adenocarcinoma who developed an excellent response after a single-dose of nivolumab. Pre-treatment tumor was found to have moderate CD3 and PD-L1 positivity by immunohistochemical staining. Evaluation of exceptional responders and non-responders are critical to furthering our understanding of the mechanisms of action (and resistance) to these agents.

## Background

In the past decade, large scale sequencing efforts unraveled the role of molecular driver events in the etiopathogenesis of non-small cell lung cancer (NSCLC), primarily in adenocarcinoma. Molecular targeted therapies designed to exploit these weaknesses have transformed the management of the minority of patients with advanced adenocarcinoma whose tumors harbor mutations in *epidermal growth factor receptor* (*EGFR*) or rearrangements in *anaplastic lymphoma kinase* (*ALK*) or *c*-*ros oncogene 1* (*ROS1*). Unfortunately, little progress has been made in the treatment of patients with the most frequently observed driver oncogene, mutant *KRAS*. *KRAS* is mutated in one-third of all malignancies and approximately 25 % of all NSCLC [1]. Further, acquired resistance to the currently targetable driver mutations is all but inevitable [2, 3]. Moreover, the prognosis of patients whose tumors do not harbor these genetic changes or those who progress on these agents continue to be treated with chemotherapy and have a median survival of 10–12 months.

Programmed death 1 (PD-1) receptor is an inhibitory T cell receptor expressed by activated T-cells and engaged by ligands PD-L1 and PD-L2 of the B7-ligand superfamily normally expressed by infiltrating immune cells in response to viral infection. PD-1/PD-L1 axis constitutes a negative regulatory mechanism by which T-cell activation is homeostatically regulated; but is hijacked by tumors to circumvent effective anti-tumor immunity [4–6]. PD-1 blockade has been explored as an immunotherapeutic strategy with resounding success in immunogenic tumors (melanoma, renal cell carcinoma, urothelial carcinoma) and additionally in tumors not formerly thought immunogenic including squamous NSCLC. Regulatory approval in squamous NSCLC was granted in early 2015 on the basis of improved survival compared to docetaxel in a 2nd line phase III study though activity has been reported in non-squamous NSCLC as well [7]. In these (and other studies) patients are typically first evaluated for response at 12 weeks. Herein, we report a patient with advanced *KRAS* mutant pulmonary adenocarcinoma treated with nivolumab after progressing on multiple therapies who had a remarkable response after just a single dose.

## Case presentation

A 77-year-old African-American female heavy current smoker presented with dyspnea in September 2012. Initial computer tomography imaging revealed bilateral upper lobe masses with mediastinal lymphadenopathy and bilateral pulmonary and pleural nodules consistent with malignancy. Biopsy revealed CK7/TTF-1 positive adenocarcinoma. Molecular studies were notable for a *KRAS* exon 2 (pG12C, c.34G>T) mutation and *TP53* (pR283P, c.848G>C) but otherwise negative for 48 other key cancer genes as determined by the Ampliseq Cancer Hotspot Panel v2 including *EGFR*/*BRAF*/*PIK3CA* mutations, *ALK*/*ROS1*/*KIF5B/RET* rearrangements and *MET* amplification.

She received 10 fractions of radiation therapy (RT) to mediastinum, then carboplatin and pemetrexed for 4 cycles with a partial response that lasted 5 months before progressing in June 2013. Between June 2013 and January 2015, she received docetaxel, an investigational FAK inhibitor, gemcitabine and pemetrexed. In January 2015, she developed non-infectious pericarditis and spinal metastases requiring RT during which systemic therapy was interrupted till May 2015. Restaging scans documented further pulmonary and mediastinal lymph node progression with no evidence of extra-thoracic metastases. She then received nivolumab 3 mg/kg on May 20, 2015. Two weeks after her first dose, she was subsequently admitted for failure to thrive and dysphagia and was found to have a severe esophageal stricture near the GE junction secondary to extrinsic compression. She was subsequently discharged to a skilled nursing facility with plan for hospice. Two weeks after discharge, a dramatic clinical improvement was observed (weight gain, resolution of dysphagia and improved performance status) and the patient was seen in clinic. Her course was subsequently complicated by exacerbation of chronic obstructive pulmonary disease (COPD) requiring admission at which time a restaging CT scan was obtained, then convalescence in a nursing home, which precluded further nivolumab administration despite a remarkable improvement in dysphagia and performance status. Restaging scans showed marked regression in dominant parenchymal lung masses and para-esophagal lymph node commensurate with improving performance status and subjective dysphagia. She has since received 3 further doses of nivolumab with ongoing response on last restaging scans on November 13, 2015.

## Discussion

**Fig. 1:**
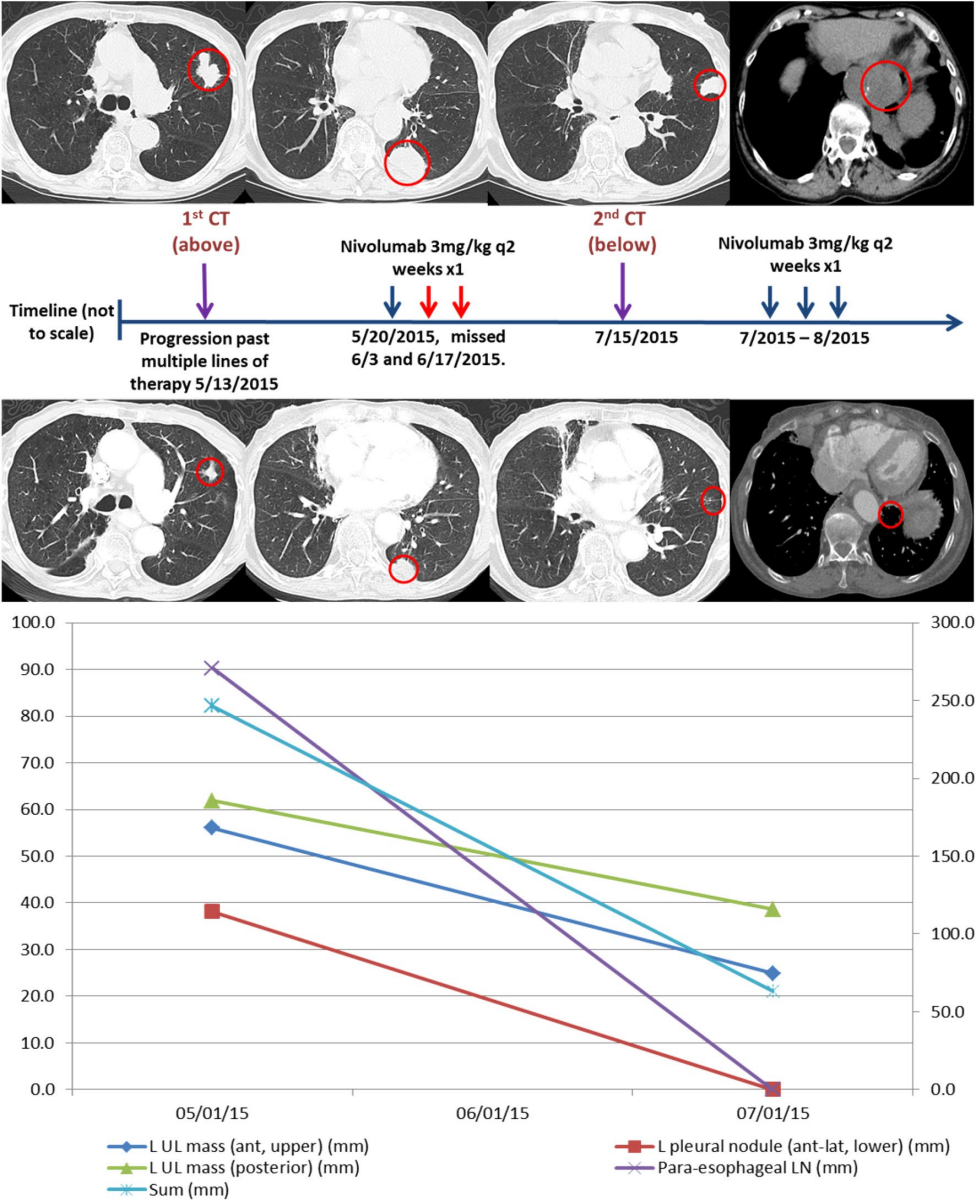
Changes in Bi-dimensional tumor measurements of target and non-target lesions. Computer tomography (CT) scans depict imaging studies done on 5/13/2015 (above) and 7/15/2015 (middle). Lower graph depicts changes in size of target and non-target lesions at both time-points. Tumors were evaluated using Response Evaluation Criteria In Solid Tumors (RECIST v1.1). Left axis depicts maximal bi-dimensional measurement for 4 (1 target and 3 non-target) lesions while right axis depicts sum of maximal bi-dimensional measurements for all 4 lesions. All measurements are in mm. Nivolumab 3 mg/kg was administered on 5/20/2015 but subsequent doses were missed given multiple admissions. Prior to resumption of therapy following multiple admissions, a restaging scan was repeated on 7/15/2015. Remarkably, all lesions had shrunk considerably with 2 non-target lesions (left pleural nodule and para esophageal lymph node) disappearing completely after a single dose of nivolumab. Patient received 3 doses of nivolumab between 7/8/2015 and 8/12/2015. Therapy is ongoing with continued response

This report highlights the significant depth and duration of responses possible in select patients treated with PD-1 inhibitor therapy (see Fig. 1). Similar dramatic responses have been observed with use of PD-1 inhibitors in other malignancies but this marks the first report of such a dramatic response in a patient with NSCLC with non-squamous histology [8].

**Fig. 2:**
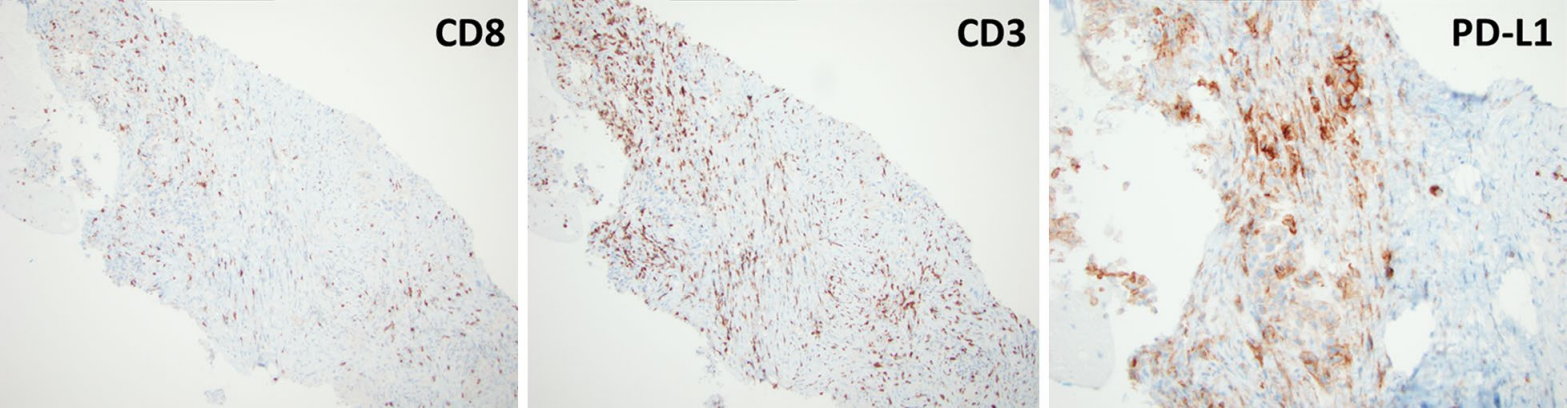
Histopathological analysis of pre-treatment tumor tissue. Pre-treatment formalin-fixed paraffin-embedded tissue specimen was stained for CD3, CD8 and PD-L1 by immunohistochemistry. Sections indicate presence of CD3+ and CD8+ T-cells though CD3+ T-cells outnumbered CD8+ T-cells. PD-L1 staining was heterogenous, localized to tumor tissue and of moderate intensity (2+, using method previously described by Taube JM et al., Sci Transl Med 2012)

Programmed death 1 (PD-1) receptor is expressed by activated T-cells and engaged by ligands PD-L1 and PD-L2. PD-1/PD-L1 axis constitutes a negative regulatory mechanism by which T-cell activation is homeostatically regulated; and is hijacked by tumors to circumvent effective anti-tumor immunity. Currently PD-1 inhibitors are approved for the treatment of BRAF V600 mutated and wild-type melanoma, advanced renal cell carcinoma and NSCLC with ongoing regulatory phase III trials in a host of other diseases including glioblastoma, head and neck carcinoma, small cell lung cancer, microsatellite unstable colorectal cancer, hepatocellular carcinoma, sarcoma, and Hodgkin’s lymphoma [9, 10]. In NSCLC, PD-1 blockade is only effective in 20–30 % of NSCLC patients with a 1-year survival rate of 42 % that declines to 18 % at 3 years [11]—much lower than in other malignancies such as melanoma and Hodgkin’s lymphoma. Predictive biomarkers of PD-1 therapy are urgently needed in general but especially in NSCLC where patients often have significant co-morbidities necessitating upfront use of the most efficacious agents. Notably, in this patient, pre-treatment tumor tissue demonstrated PD-L1 and CD3 staining of moderate intensity suggesting a possible explanation for the outstanding response observed (see Fig. 2).

PD-1/PD-L1 staining is highly contextual and depends on the disease and treatment setting. Evaluation of PD-1/PD-L1 staining in archival samples indicates that staining varies widely in solid tumors being highly expressed in certain tumors (melanoma, sarcoma) but minimally in others (hepatocellular carcinoma) [12]. Within tumors, PD-1/PD-L1 expression appears to be prognostic—being associated with poorer prognoses in multiple tumor types including testicular germ cell tumors and prostate cancer [13, 14]. The observation of PD-L1 upregulation on leukemic cells in patients on treatment with bi-specific antibodies likely represents development of a T-cell-induced immune escape mechanism—and suggests that PD-1/PD-L1 inhibition may be complementary to these therapies in this setting [15, 16]. It is likely that the most accurate biomarkers will incorporate elements that reflect the dynamic and complex nature of the immune system including tumor mutation burden and T-cell infiltrate [17, 18]. Close interrogation of the host, tumor and tumor microenvironment in both extraordinary responders and primary non-responders to PD-1 therapy will be critical to this effort.

## Consent

Written informed consent for the publication of details, laboratory results and images relating to individual participants was obtained from the participants for publication of this case report and any accompanying images. Copies of the written consents are available for review by the Editor-in-Chief of this journal.

## Abbreviations

NSCLC: non-small cell lung cancer
EGFR: epidermal growth factor receptor
ALK: anaplastic lymphoma kinase
ROS1: c-ros oncogene 1
PD-1: programmed death 1
RT: radiation therapy
COPD: chronic obstructive pulmonary disease
